# Supplementary material for: Effective and Rapid Generation of Functional Neutrophils from Induced Pluripotent Stem Cells Using ETV2-Modified mRNA
Source: Stem Cell Reports. 2019 Nov 7;13(6):1099–110. doi: 10.1016/j.stemcr.2019.10.007 (PMC6915846; doi:10.1016/j.stemcr.2019.10.007)
Supplement: Document S1. Figures S1 and S2 and Table S1 [file mmc1.pdf]

Stem Cell Reports, Volume *13*

Supplemental Information

Effective and Rapid Generation of Functional Neutrophils from Induced Pluripotent Stem Cells Using ETV2-Modified mRNA

Vera S. Brok-Volchanskaya, David A. Bennin, Kran Suknuntha, Lucas C. Klemm, Anna Huttenlocher, and Igor Slukvin

Supplementary Information

V.S. Brok-Volchanskaya, D. A. Bennin, K. Suknuntha, L. Klemm, A. Huttenlocher, and I. Slukvin

Effective and rapid generation of functional neutrophils from induced pluripotent stem cells using ETV2 modified mRNA

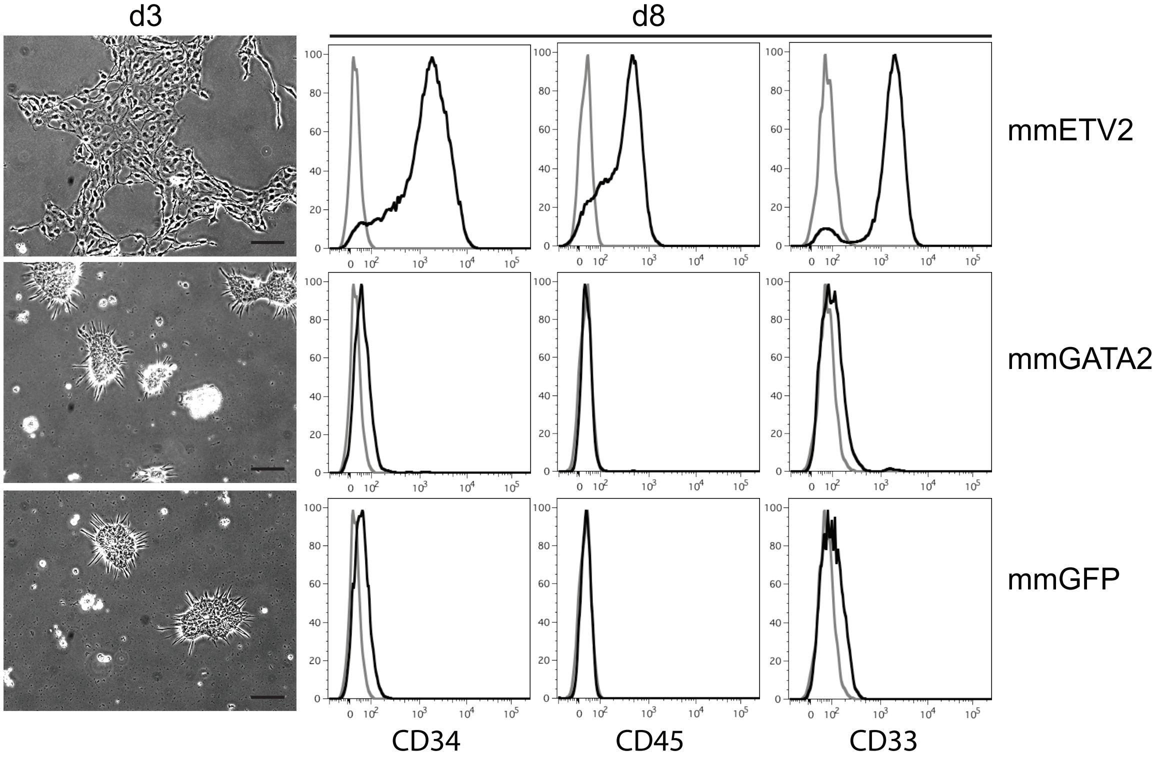

Supplementary Figure 1. Induction of myeloid progenitors is specific for ETV2 modified mRNA. iPSCs were transfected with ETV2, GATA2 or GFP modified RNA and cultured as outlined in Fig. 1A. Formation of $CD34^{+}CD33^{+}CD45^{+}$ myeloid progenitors was observed only in ETV2 mmRNA transfected cultures.

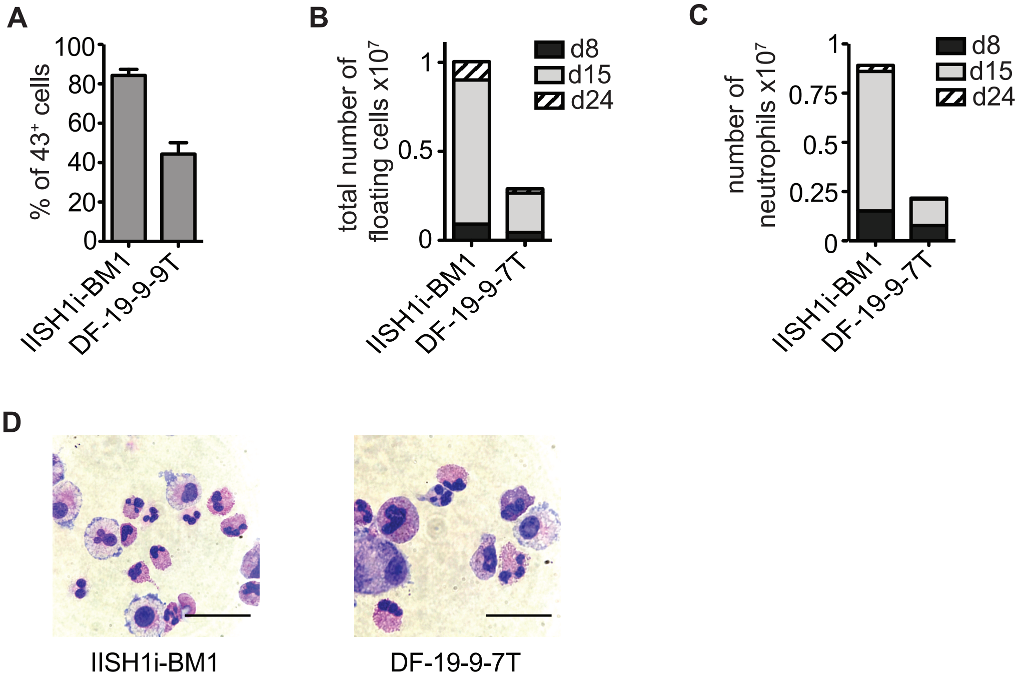

Supplementary Figure 2. Neutrophil production from IISH1i-BM9 and DF-19-7T iPSC lines using ETV2 modified mRNA. (A) Proportion of $CD43^+$ cells formed on day 8 of differentiation. (B) Total number of floating cells collected from differentiation cultures. (C) Total number of neutrophils produced in cultures. (D) Cytospins showing the morphology of the generated neutrophils.

Supplementary Video 1-4

Video 1-Fig 4a hiPSC derived neutrophils random migration.

Video 2-Fig 4a hiPSC derived neutrophils chemotaxing to fMLP.

Video 3-Fig 4a hiPSC derived neutrophils chemotaxing to IL8.

Video 4-Fig 4c hiPSC derived neutrophils migrating and phagocytosing *Pseudomonas aeruginosa.*

Supplementary Table 1. Antibodies used in this study.

**Antigen**	**Conjugate**	**Source**	**Cat No.**
CD10	BV510	BD Biosciences	563032
CD11b	APC	BD Biosciences	550019
CD11b	PE	Life Technologies	12-0118-42
CD15	BV711	BD Biosciences	563142
CD16	FITC	Life Technologies	11-0168-42
CD16	APC	Life technologies	17-0168-42
CD16	PE-Cy7	BD Biosciences	557744
CD33	PE-Cy5	Biolegend	303406
CD34	PE-Vio770	Miltenyi Biotec	130-100-844
CD34	FITC	BD Bioseceinces	555821
CD41a	APC	BD Bioseceinces	559777
CD43	VioBlue	Miltenyi Biotec	130-097-373
CD45	PE	Miltenyi Biotec	130-113-118
CD45	PE-Vio770	Miltenyi Biotec	130-110-634
CD45	APC-Vio770	Miltenyi Biotec	130-110-773
CD66b	PE	BD Biosciences	561650
CD144	PE-Vio770	Miltenyi Biotec	130-100-720
CD182	PE	Invitrogen	12-1829-42
CD235a	APC	BD Bioseceinces	551336
Lactoferrin	PE	Life Technologies	GIC206
MPO	FITC	Invitrogen	11-1299-42
Ghost red	780	Tonbo Biosciences	13-0865-T100
