# Supplementary material for: Effective and Rapid Generation of Functional Neutrophils from Induced Pluripotent Stem Cells Using ETV2-Modified mRNA
Source: Stem Cell Reports. 2019 Nov 7;13(6):1099–110. doi: 10.1016/j.stemcr.2019.10.007 (PMC6915846; doi:10.1016/j.stemcr.2019.10.007)
Supplement: Document S2. Article plus Supplemental Information [file mmc6.pdf]

Stem Cell Reports
Resource
OPEN ACCESS

Effective and Rapid Generation of Functional Neutrophils from Induced Pluripotent Stem Cells Using ETV2-Modified mRNA

Vera S. Brok-Volchanskaya,[1] David A. Bennin,[2] Kran Suknuntha,[1,3,4] Lucas C. Klemm,[2] Anna Huttenlocher,[2,6] and Igor Slukvin[1,3,5,6,*]
[1]Wisconsin National Primate Research Center, University of Wisconsin, Madison, WI 53715, USA
[2]Departments of Pediatrics and Medical Microbiology and Immunology, University of Wisconsin-Madison, Madison, WI 53706, USA
[3]Department of Pathology and Laboratory Medicine, Wisconsin National Primate Research Center, University of Wisconsin, 1220 Capitol Court, Madison, WI 53715, USA
[4]Department of Pharmacology, Faculty of Science, Mahidol University, Bangkok 10400, Thailand
[5]Department of Cell and Regenerative Biology, University of Wisconsin School of Medicine and Public Health, Madison, WI 53707-7365, USA
[6]Co-senior author
*Correspondence: islukvin@wisc.edu
https://doi.org/10.1016/j.stemcr.2019.10.007

SUMMARY

Human induced pluripotent stem cells (hiPSCs) can serve as a versatile and scalable source of neutrophils for biomedical research and transfusion therapies. Here we describe a rapid efficient serum- and xenogen-free protocol for neutrophil generation, which is based on direct hematoendothelial programming of hiPSCs using ETV2-modified mRNA. Culture of ETV2-induced hematoendothelial progenitors in the presence of GM-CSF, FGF2, and UM171 led to continuous production of generous amounts of CD34$^+$CD33$^+$ myeloid progenitors which could be harvested every 8–10 days for up to 30 days of culture. Subsequently, myeloid progenitors were differentiated into neutrophils in the presence of G-CSF and the retinoic acid agonist Am580. Neutrophils obtained in these conditions displayed a typical somatic neutrophil morphology, produced reactive oxygen species, formed neutrophil extracellular traps and possessed phagocytic and chemotactic activities. Overall, this technology offers an opportunity to generate a significant number of neutrophils as soon as 14 days after initiation of differentiation.

INTRODUCTION

Despite recent advances in prevention and management, myelosuppression and febrile neutropenia remains one of the most disturbing complications of cancer therapies and common cause of morbidity and mortality, especially in pediatric cancer patients (Castagnola et al., 2007; Lyman and Poniewierski, 2017). In pioneering studies, correcting neutropenia with granulocyte transfusion was shown to be efficient in the clinical management of septicemia (Graw et al., 1972). Although recent clinical trials produced inconclusive evidence concerning its efficiency, granulocyte transfusion is still perceived as a life-saving support in neutropenic patients, especially in pediatric patients resistant to antimicrobial therapies (Gea-Banacloche, 2017; Gurlek Gokcebay and Akpinar Tekgunduz, 2018; Valentini et al., 2017; West et al., 2017). However, the complicated logistics of granulocyte collection, the need for pre-treating donors with granulocyte colony-stimulating factor (G-CSF) or steroids, difficulties in collecting a sufficient number of good quality granulocytes and the limited storage time (24 h); all hamper the utility of granulocyte transfusion for correcting neutropenia, and may contribute to the inconclusive results observed in clinical trials.

Human induced pluripotent stem cells (hiPSCs) offer the potential to serve as a versatile and scalable source of granulocytes. Although our group and others have demonstrated the feasibility of neutrophil generation from hiPSCs (Choi et al., 2009a; Lachmann et al., 2015; Saeki et al., 2009; Sweeney et al., 2016; Trump et al., 2019), previously described methods rely on the use of serum, feeder, or embryoid body formation, which hampers their adoption for good manufacturing practice (GMP) standards and clinical use. In present studies, we have developed a three-step protocol for efficient neutrophil production from hiPSCs in 2D serum- and feeder-free conditions using direct programming with modified mRNA (mmRNA), which is less immunogenic and more stable form of mRNA (Badieyan and Evans, 2019; Suknuntha et al., 2018). Initially, hiPSCs are directly programmed into hematoendothelial progenitors using ETV2 mmRNA, which then differentiated into myeloid progenitors in the presence of granulocyte-macrophage (GM)-CSF, fibroblast growth factor 2 (FGF2), and UM171. Myeloid progenitors could be continuously collected from cultures every 8–10 days for up to 30 days after ETV2 transfection, and subsequently differentiated into mature neutrophils in the presence of G-CSF and the retinoic acid agonist Am580. This method significantly expedites generation of neutrophils, with the first batch of neutrophils available as soon as 14 days after initiation of differentiation and allows the generation of up to 1.7 × 10^7 neutrophils from 10^6 hiPSCs. The proposed differentiation system may be suitable for generating mature functional granulocytic cells for correction of neutropenia.

© 2019 The Author(s). This is an open access article under the CC BY license (http://creativecommons.org/licenses/by/4.0/).

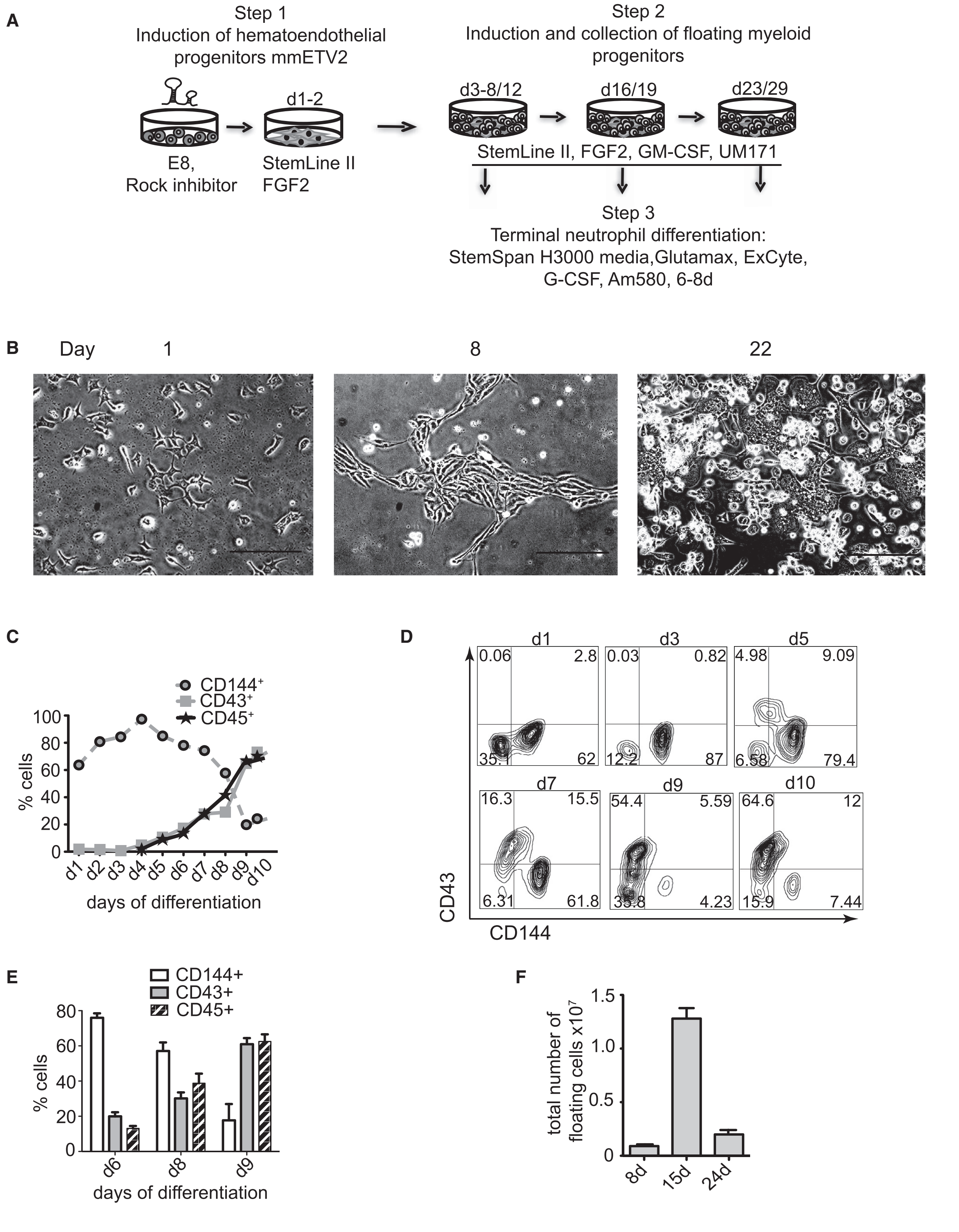

(legend on next page)

When coupled with genetic engineering technologies, this protocol can be also used to interrogate the role of genes involved in neutrophil development and function.

RESULTS

Induction of Hematoendothelial Program with Myeloid Potential by ETV2 mmRNA

Previously, we demonstrated that overexpressing transcription factors ETV2 and GATA2 is sufficient to induce a pan-myeloid program in hiPSCs, which proceeds through a hemogenic endothelium (HE) stage (Elcheva et al., 2014). Although we have found that constitutive overexpression of ETV2 using lentiviral vectors induces predominantly non-hemogenic endothelium, we also noted that ETV2 induces GATA2 expression in hPSCs and very few HE with macrophage potential (Elcheva et al., 2014). In addition, our recent studies suggest that molecular mechanisms upstream of GATA2 are sufficient to specify hematoendothelial programs in hPSCs, while GATA2 is required for endothelial-to-hematopoietic transition (Kang et al., 2018). Given these findings and studies demonstrating the critical role of ETV2 threshold for hematoendothelial commitment (Zhao and Choi, 2017) and obligatory downregulation of ETV2 during subsequent stages of hematopoietic development in the embryo (Hayashi et al., 2012), we explored whether transitional expression of ETV2 with mmRNA alone is sufficient for hematoendothelial programming in hiPSCs. For ETV2 mmRNA production we used a transcription template which contains a single 5′ UTR and a single 3′ UTR from the β globin gene. In previous studies, we found that mmRNA in this configuration provide maximum protein levels in hPSCs (Suknuntha et al., 2018). Overexpression of mmETV2 following culture of transfected hiPSCs in Stemline II serum-free medium with FGF2, rapidly induces CD144$^+$-expressing endothelial cells that, on addition of GM-CSF, form floating CD43$^+$ blood cells, most of which co-express CD45 (Figures 1A–1E). Transfection of cells with GFP or GATA2 mmRNA could not induce hematopoietic programming in these conditions, indicating that this effect is specific for ETV2 and is not an artifact of the mmRNA transfection procedure (Figure S1). Interestingly, as soon as the first floating cells appeared (starting on day 5 after ETV2 treatment), some of them began to adhere and continue producing floating blood cells for another 2 weeks (Figure 1B), thereby allowing for continuous collection of blood cells for up to 3 weeks of culture. Typically, around 0.9×10^6, 1.25×10^7, and 2×10^6 floating blood cells can be collected after the first, second, and third weeks, accordingly from 10^6 hiPSCs transfected with ETV2 (Figure 1F).

CD45$^+$ cells generated in ETV2 mmRNA-induced cultures co-expressed CD34 and lacked expression of megakaryocytic and erythroid markers CD41 and CD235a (Figure 2A). They quickly acquired early myeloid progenitor marker CD33 with more than 80% of total cells in suspension were positive for CD34 and CD33 on days 5–8 of differentiation (Figures 2B and 2C). However, CD33 marker was gradually decreased while mature myeloid markers CD11b and CD16 gradually increased if floating cells are collected at later time points (Figure 2D). Collected floating cells displayed myeloid progenitor morphology on cytospins (Figure 2E) and possess GM- and M-colony-forming cell (CFC) potential (Figure 2F), thereby suggesting that the ETV2-induced program was mostly restricted to myelomonocytic cells. Kinetic analysis revealed that CFC potential of myeloid progenitors greatly increased from day 4 to 9, but gradually decreased afterward (Figure 2F).

Next, we investigated the cytokine and growth factor requirements for optimal expansion of myeloid progenitors induced with ETV2. The presence of GM-CSF, which we identified as a the most critical factor for hPSC-derived myelomonocytic cells (Choi et al., 2009a, 2009b; Slukvin et al., 2006), was necessary for efficient production of myeloid progenitors because its removal substantially decreased the number of floating hematopoietic cells in cultures (Figure 2G). Similarly, withdrawal of FGF2 reduced blood production in ETV2-induced cultures (Figure 2G). To test whether myeloid cell production can be improved with the use of other cytokines or small molecules, we tested the effects of FLT3L, SCF, and small-molecule UM171, which has been shown to stimulate expansion of human cord blood CD34$^+$ cells *ex vivo* (Fares et al., 2014) and hPSC-derived myeloid progenitors enriched in G-CFCs (Mesquitta et al., 2019). We have found that the presence of SCF and

Figure 1. Induction of a Hematoendothelial Program Following Transfection hiPSCs with ETV2 mmRNA

(A) Schematic diagram of optimized protocol used for generation of neutrophils from hiPSCs in serum-, xeno-, and feeder-free conditions.

(B) Representative light microscopy images showing hematoendothelial development following transfection IISH2i-BM9 hiPSCs with ETV2 mmRNA and culture with FGF2 and GM-CSF. Scale bars, 300 μm.

(C and D) Representative experiment showing kinetics of CD144 and CD43 expression (percentages (C) and flow cytometry dot plots (D) in cultures.

(E) Expression of CD144, CD43, and CD45 on day 6, 8, and 9 after ETV2 transfection. Bars show mean ± SE for 3 independent experiments.

(F) Yield of floating myeloid cells in step 2 cultures from 10^6 undifferentiated hiPSCs. Bars show mean ± SE for 2 independent experiments. See also Figure S1.

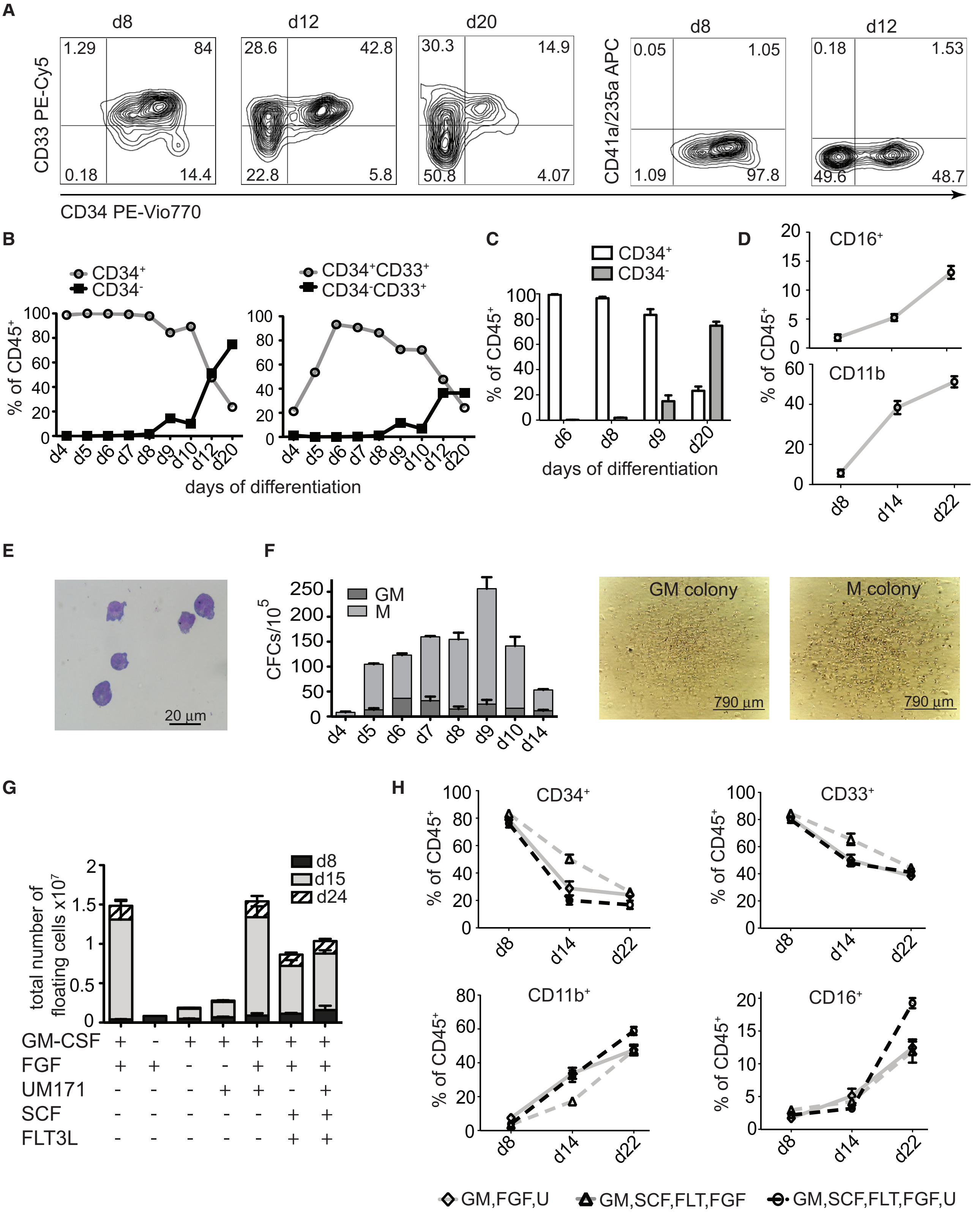

(legend on next page)

FLT3L slightly decreased the number of collected floating cells during differentiation, while UM171 had no significant effect on the number of hematopoietic cells. Flow cytometric analysis revealed no significant effect of studied cytokines and small molecules on myeloid cell phenotype in cultures (Figure 2H). Thus, we concluded that FGF2 and GM-CSF are the two most critical cytokines to support myeloid lineage development in ETV2 mmRNA-transfected hiPSCs.

Induction of Neutrophils from ETV2-Induced Myeloid Progenitors

To induce formation of neutrophils from myeloid progenitors, we cultured them in StemSpan H3000 medium with G-CSF and retinoic acid agonist Am580, which is known to promote neutrophil production from human somatic $CD34^+$ cells (Li et al., 2016). After 7 days of culture in these conditions, we observed formation of cells with typical neutrophil phenotype and morphology (Figures 3A and 3B). Although myeloid progenitors produced some macrophages, they were adherent to the plate while the collected floating cells contained a population of highly enriched in neutrophils (Figure 3B). Phenotypic analysis revealed that most of the collected floating cells expressed CD11b, MPO, and CD182, and greater than 50% were CD16-positive and expressed lactoferrin. However, generated neutrophils expressed relatively low levels of CD66b and were lacking the CD10 marker, which are typically present on mature peripheral blood neutrophils (Figure 3A). Although the effect of UM171 on the output of myeloid progenitors in step 2 differentiation cultures was minimal, we noticed that cells from UM171-treated cultures generated much higher neutrophils in the final differentiation step compared with myeloid progenitors generated in cultures without UM171 (Figure 3C). As mentioned previously, following collection of floating cells from step 2 differentiation cultures, adherent cells continued to generate myeloid progenitors that could be collected for an additional 2 weeks. Although the number of floating cells increased more than 10-fold following the second collection (second week; Figure 1F), they produce fewer neutrophils as compared with myeloid progenitors collected at day 8 of differentiation (Figure 3D). During the third week of culture, the number of floating myeloid cells collected dramatically decreased, although they were still able to differentiate into neutrophils. Overall, combining total neutrophil output from myeloid progenitor cultures collected over a 3-week period, we were able to generate up to 1.7×10^7 neutrophils from 10^6 hiPSCs.

Functional Characterization Obtained from ETV2-Induced Myeloid Progenitors

Functional analysis revealed that ETV2-induced neutrophils phagocytose pHrodo *E. coli* particles, although we noticed the presence of a population of immature myeloid progenitors lacking phagocytic activity in *ex-vivo*-generated cellular products (Figure 3E). In addition, ETV2-induced neutrophils generated reactive oxygen species in response to treatment with phorbol esters (PMA) (Figure 3F) and demonstrated chemotactic migration to N-formyl-methionyl-leucyl-phenylalanine (fMLP) and interleukin-8 (IL-8) using microfluidic analysis (Figures 4A and 4B; Videos S1, S2, and S3). Although, the chemotactic index is not as robust as compared with primary human neutrophils, ETV2-derived neutrophils demonstrated directed migration to both IL-8 and fMLP and improved chemotactic responses as compared with the neutrophil-like PLB-985 cells (Cavnar et al., 2012; Powell et al., 2017). Finally, the ETV2-induced neutrophils also chemotaxed to and phagocytosed the live bacteria *Pseudomonas aeruginosa* (Figure 4C; Video S4) and formed an extracellular fibril matrix composed of granule protein and chromatin, characteristic of neutrophil extracellular traps (NETs) on treatment with phorbol ester (100 nm PMA; Figure 5). Taken together, these findings demonstrate that ETV2-induced neutrophils demonstrate phagocytic, chemotactic, and signaling functions similar to primary human neutrophils.

Figure 2. Formation of Myeloid Progenitors in hiPSC Cultures Transfected with ETV2 mmRNA

(A) Flow cytometric analysis of CD34, CD33, and CD235a/CD41a expression in ETV2 mmRNA-transfected IISH2i-BM9 cultures differentiated in the presence of GM-CSF and FGF2.

(B) Representative experiment shows kinetics of CD34 and CD33 expression.

(C) Bar graphs show proportion of $CD34^+$ and $CD34^-$ cells in floating fraction at indicated days of differentiation. Results are mean + SE for 3 experiments.

(D) Kinetics of CD11b and CD16 expression in cells collected from step 2 cultures. Error bars are + SE for 3 experiments.

(E) Cytopsin showing the morphology of the myeloid progenitors generated in cultures after 10 days of differentiation. Scale bar, 20 μm.

(F) Colony-forming potential of myeloid progenitors formed in cultures. Bars show mean ± SE for 2 independent experiments performed in duplicates. Representative images of colonies formed by cells collected from step 2 cultures are shown. Scale bars, 790 μm.

(G) Yield of floating cells from IISH2i-BM9 hiPSCs in the presence of different cytokine combinations and UM171. Bars show mean ± SE for 3 (GM-CSF, FGF, and UM171) or 2 (remaining combinations) independent experiments.

(H) Effect of different cytokine combinations on phenotype of myeloid cells. GM is GM-CSF, FGF is FGF2, FLT is FLT3L, U is UM171. Error bars are ± SE for 3 experiments.

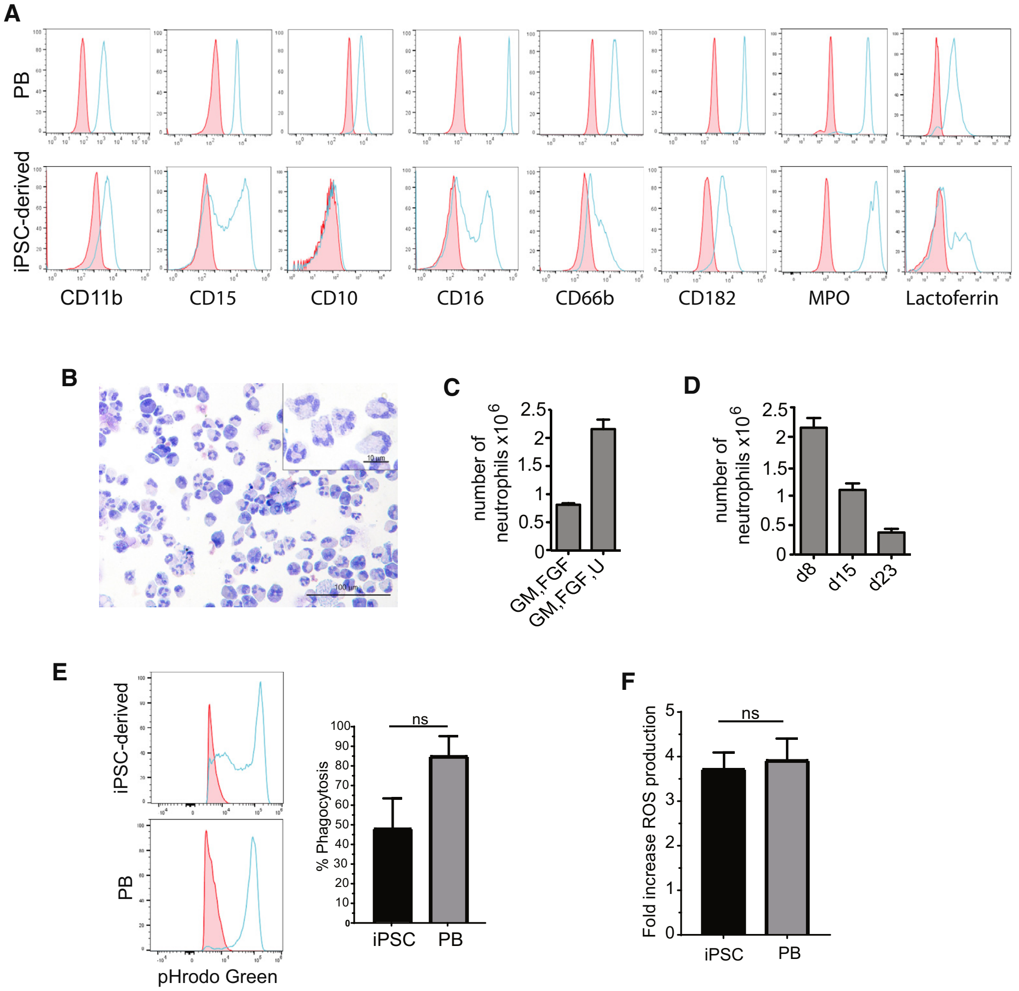

Figure 3. Induction of Neutrophil Formation from Myeloid Progenitors

(A) Flow cytometric analysis of generated neutrophils. Plots show unstained control (red) and specific antibody (blue) histograms.

(B) Cytospin showing the morphology of the generated neutrophils. Scale bars, 100 μm and 10 μm (insert).

(C) Neutrophil yields from 10^6 myeloid progenitors that were cultured with or without UM171 and collected on day 8 after ETV2 mmRNA transfection. Bars show mean ± SE for 3 (GM, FGF, and U) and 2 (GM and FGF) independent experiments.

(D) Total number of neutrophils obtained from 10^6 myeloid progenitors that were cultured with UM171 and collected at different days of after ETV2 mmRNA transfection. Bars show mean ± SE for 2 independent experiments.

(E) Phagocytosis of pHrodo Green *E. coli* particles by neutrophils generated from IISH2i-BM9 hiPSCs. Solid red peaks on flow graphs are control cells incubated on ice with bio-particles, blue traces are cells containing acidified, fluorescent *E. coli* bio-particles from 37°C incubation. Bar graph is from 3 independent experiments showing percent of cells from 37°C incubation with phagocytosed acidified *E. coli* bio-particles. Bars show mean ± SE. Difference between iPSC and primary neutrophils is not statistically significant ($p = 0.3134$) as determined by unpaired t test.

(F) Reactive oxygen species production of hiPSC-derived neutrophils compared with primary blood neutrophils at 90 min. Bar graph is from 3 independent experiments showing fold increase of 50 ng/mL PMA-treated cells over control-treated cells. Bars show mean ± SE. Difference between hiPSC versus primary neutrophils is not statistically significant ($p = 0.7522$) as determined by unpaired t test.

See also Figure S2.

Comparative Analysis of the Efficacy of the ETV2 mmRNA-Based Protocol for Neutrophil Induction from Different hiPSC Lines

Two more hiPSC lines, bone marrow-derived iPSC (IISH1i-BM1) and fibroblast-derived iPSC (DF-19-9-7T) lines were assessed for their capacity of inducing neutrophils differentiation using ETV2 mmRNA. Both cell lines were successfully programmed to myeloid progenitors, although with different efficiencies. Thus, for IISH1i-BM1 and DF-19-9-7T cell lines programming efficiency to $CD43^+$ cells were

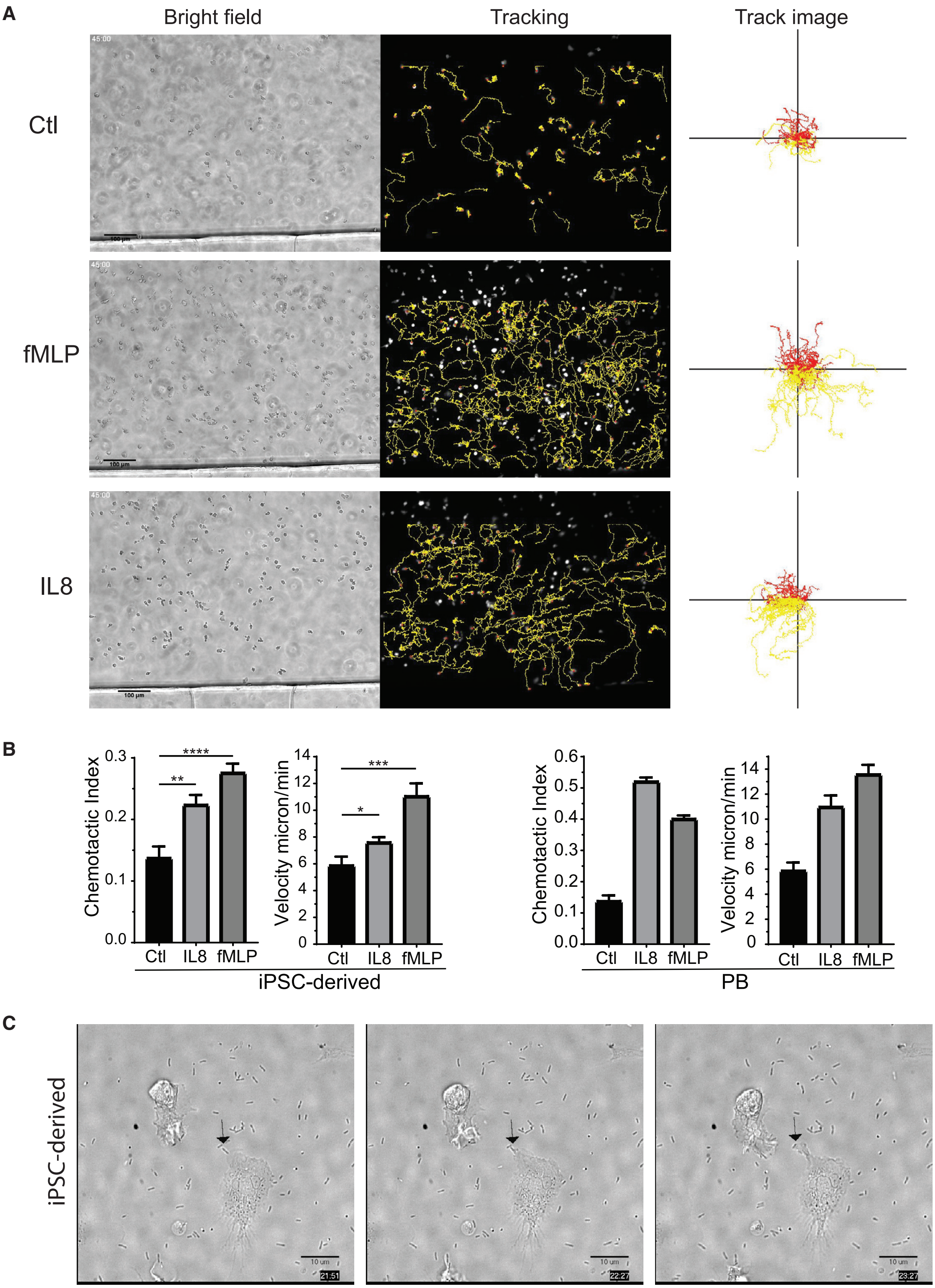

(legend on next page)

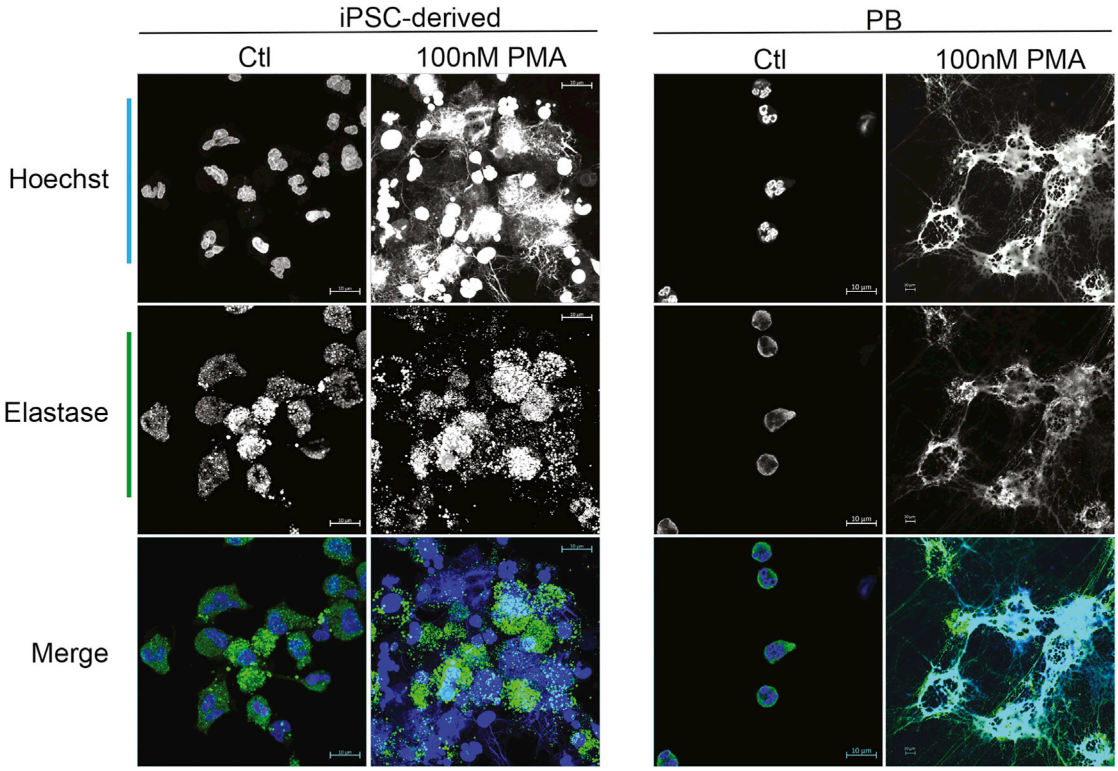

Figure 5. Neutrophil Extracellular Trap Formation
Maximum intensity projections of hiPSC and primary blood (PB) neutrophils. Both show characteristic multi-lobed nuclei in blue and the azurophilic granule protein neutrophil elastase in green with control treatment. On treatment with 100 nM PMA both hiPSC and primary blood neutrophils form an extracellular fibril matrix composed of granule protein and chromatin, characteristic of neutrophil extracellular traps. Scale bars, 10 μm.

84% and 44%, respectively. After terminal differentiation step, neutrophils exhibited the expected morphology (Figure S2).

DISCUSSION

In this study we developed a simple and efficient method for generating neutrophils from hiPSCs by triggering the myeloid hematoendothelial program with ETV2 mmRNA, subsequently inducing and expanding myeloid progenitors, and finally differentiating them into neutrophils. This methods allows for neutrophil production in GMP-compatible conditions without feeder cells, or serum and xenogenic components. The first batch of neutrophils can be obtained within a very short period of time (14 days after hiPSC transfection with ETV2 mmRNA). Because myeloid progenitors generated

Figure 4. Analysis of Migratory Potential of hiPSC-Derived Neutrophils
(A) Images from 45-min time-lapse movies of hiPSC-derived neutrophils during chemotaxis to vehicle control, fMLP, or IL-8 on fibrinogen. Yellow lines showing automated cell tracking. Scale bars, 100 μm.
(B) Quantification of chemotactic index and velocity of hiPSC neutrophils, showing significant differences of hiPSC neutrophils exposed to chemoattractant compared with treatment with vehicle control. p values for chemotactic index, control to IL-8 $p = 0.0040$, control to fMLP $p < 0.0001$. p values for velocity, control to IL-8 $p = 0.0404$, control to fMLP $p = 0.0009$ as determined by unpaired t test. Primary blood neutrophils showing chemotactic and velocity values. Bars show mean ± SE from 3 independent experiments with technical replicates, but not biological replicates for statistical analysis.
(C) Montage image from high-resolution time-lapse movie of hiPSC neutrophils migrating and phagocytosing *Pseudomonas aeruginosa*. Arrow indicates one point during the movie when the cell is targeting bacteria. Scale bars, 10 μm. All functional assays with hiPSC-derived neutrophils were performed on batches obtained from myeloid progenitors collected at 8 or 12 days after ETV2 transfection.
See also Videos S1, S2, S3, and S4.

from ETV2 mmRNA-transfected cells continue generating neutrophil precursors for up to 3 weeks of culture in the presence of GM-CSF, FGF2, and UM171, they can be collected weekly and used to produce neutrophils. This allows for total collection of up to 1.7×10^7 neutrophils from 10^6 hiPSCs. The method described here is based on a 2D culture system and is easily amendable to robotic manufacturing. Although neutrophils produced by this method had the capacity to produce reactive oxygen species, migrate in response to fMLP and IL-8, phagocytose bacteria, and form NETs, they were somewhat different from peripheral blood neutrophils and neutrophils produced from hPSCs in the presence of serum and feeders in our previous studies (Choi et al., 2009a). They had unique CD10-negative CD66b^{low} phenotype and displayed somewhat reduced *E. coli* phagocytosis activity. CD10 is expressed in segmented neutrophils. However, CD10 expression is reduced in peripheral blood neutrophils from newborn infants or persons treated with G-CSF (Penchansky et al., 1993; Zarco et al., 1999), while neutrophil activation with GM-CSF, tumor necrosis factor, or complement component 5a increases CD10 expression (Kuijpers et al., 1991; Werfel et al., 1991). Thus, it is possible that lack of CD10 expression may be associated with the unique serum-free conditions we are using for their differentiation. It has become clear that, in addition to their phagocytic activities and their role in innate host defense, neutrophils contribute to the regulation of immune responses (reviewed in Scapini et al., 2016). Further detailed analysis of the function of hiPSC-derived neutrophils generated in our conditions is essential to determine whether their unique phenotype is associated with proinflammatory or immunosuppressive properties. Nevertheless, these cells will also provide a powerful tool to analyze pathways that regulate neutrophil function since, unlike primary human neutrophils, they are amenable to genetic manipulation and modeling of human disease mutations.

Successful generation of myeloid progenitors in our studies was achieved with ETV2 mmRNA. ETV2 belongs to the ETS family of transcription factors and is recognized as a master regulator of hematoendothelial fate, which is transiently expressed in specifying hematoendothelial progenitors (Garry, 2016; Kataoka et al., 2011; Sumanas and Choi, 2016). ETV2 directly induces genes required for specification of hematopoietic and vascular cells including other ETS genes and GATA2 (reviewed in Sumanas and Choi, 2016). In our present studies, we revealed that transient transfection of hiPSCs with ETV2 is sufficient to induce hematoendothelial progenitors that can be subsequently differentiated to neutrophils. Compared with classical differentiation methods, direct ETV2-mediated programming proceeds without transition through the mesodermal stage and requires a minimal numbers of growth factors (FGF2, GM-CFC, and G-CSF) to achieve neutrophil differentiation, thereby allowing for cost-efficient production of neutrophils. Although some additional developmental work is required to improve functionality of generated cells and adopt this protocol to bioreactor platform to enable clinical translation, the described method in conjunction with CRISPR/Cas9 gene editing technologies can already be used for disease modeling and interrogation of molecular mechanisms involved in neutrophil development and function.

EXPERIMENTAL PROCEDURES

Cell Culture

Bone marrow-derived IISH2i-BM9 and IISH1i-BM1 hiPSCs (Hu et al., 2011) and fibroblast-derived DF-19-9-7T hiPSCs (Yu et al., 2009) were obtained from WiCell (Madison, WI). hiPSCs were cultured on Matrigel-coated tissue culture plates in E8 medium (STEMCELL Technologies, Vancouver, Canada) (Chen et al., 2011).

mmRNA Synthesis and Transfection

Human ETV2 transcript variant 1 (NM_014209.3) was cloned into a 5′-MCS-1β construct as described previously (Suknuntha et al., 2018). To generate IVT templates with a 180-A tract, a reverse primer containing 180 T base pairs and an ATCGGTGCGGGCCTCTTCGCTA forward primer including T7 promoter were used in a PCR reaction. All PCR reactions were carried out using Phusion (Thermo Fisher Scientific). The mmRNA was synthesized using the MEGAscript T7 Kit (Ambion, Austin, TX), using a custom ribonucleoside cocktail comprised of 3′-0-Me-m7G(5′)ppp(5′)G ARCA cap analog, pseudouridine triphosphate (TriLink BioTechnologies, San Diego, CA), ATP, guanosine triphosphate, and cytidine triphosphate. The synthesis reactions were set up according to the manufacturer's instructions. Reactions were incubated for 2 h at 37°C and treated with DNAse. RNA was purified using a PureLink RNA Mini Kit (Thermo Fisher Scientific) and adjusted with RNase-free water to 100 ng/μL working concentration before being stored at −80°C. Undifferentiated hiPSCs were transfected with using TransIT-mRNA reagent in E8 medium containing ROCK inhibitor (Suknuntha et al., 2018). In brief, for transfection, single-cell suspension was prepared using HyQtase (Thermo Fisher Scientific). Per one well of transfection, a total of 2×10^5 cells in 1 mL complete E8 medium with 10 μM ROCK inhibitor (STEMCELL Technologies) were plated into a collagen IV-coated 6-well plate; 30–60 min later, a mixture of 200 ng ETV2:TransIT-mRNA (Mirus Bio, Madison, WI) was added to each well according to the manufacturer's instructions.

Feeder-, Xeno-, and Serum-free Generation of Neutrophils from hiPSCs

The day after transfection (day 1), the medium was changed with 1 mL of Stemline II (Sigma) supplemented with 20 ng/mL of human FGF2 (PeproTech). On day 2, 1 mL of the same medium was added. On day 3, the medium was changed and 1 mL of Stemline II supplemented with FGF2 (20 ng/mL), GM-CSF (25 ng/mL)

(PeproTech), and UM171 (50 nM; Xcess Biosciences) were added. This medium was added daily up to days 8 to 12. On days 8 to 12, floating cells were gently harvested and used for terminal neutrophil differentiation. Following the first collection of floating cells, 2 mL of StemlineII supplemented with FGF2, GM-CSF, and UM171 was added to the remaining adherent cells. An additional 2 mL of the same medium was added every 2 days until the next round of floating cell collection on days 16 to 19. A similar procedure was followed for the third round of cell collection on days 23 to 29. To induce neutrophil terminal differentiation, floating cells were cultured in StemSpanH300 medium (STEMCELL Technologies), supplemented with GlutaMAX 100X (Thermo Fisher Scientific), ExCyte 0.2% (Merck Millipore), human G-CSF (150 ng/mL; Amgen), Am580 retinoic acid agonist 2.5 μM (Sigma-Aldrich), and gentamycin (1,000×) (Life Technologies) at 5×10^5 cells/mL density. After 4 days, 2 mL of the same medium with all components and cytokines was added on the top of existing culture. Mature neutrophils were gently harvested from the supernatant after 6–8 days of culture, leaving the adherent macrophages, and filtered through a 70-μM mesh (Falcon, Life Sciences) before analysis.

Flow Cytometry

To analyze cell surface markers, 5×10^5 cells were stained in fluorescence-activated cell sorting buffer with the appropriate antibodies (Table S1). Ghost Dye (Tonbo Biosciences, San Diego, CA) was used to analyze the live cell population. For intracellular staining, cells were resuspended in Fixation Reagent (Medium A; Invitrogen) and incubated at room temperature for 15 min. After washing cells with PBS, cells were resuspended in 100 μL of Permeabilization Reagent (Medium B) (Invitrogen) and stained with Lactoferrin or MPO antibody (Table S1) for 15 min. Cells were analyzed using a MACSQuant Analyzer 10 (Miltenyi Biotec, San Diego, CA) or Thermo Fisher Scientific Attune and FlowJo software (Tree Star, Ashland, OR).

Wright-Giemsa Staining

To assess the morphology of cells within colonies, cells were fixed on glass slides using a Cytospin centrifuge (Cytospin 2; Thermo Shandon), stained with Wright-Giemsa solution (Sigma-Aldrich), and then observed under a light microscope (Olympus, Tokyo).

CFC Assay

Total cells were collected daily from days 4 to 10, and floating cells from day 14 cultures were subjected to hematopoietic clonogenic assay. Colony assays were performed using serum-containing MethoCult H4435 (STEMCELL Technologies) in 35-mm low-adherent culture dishes (STEMCELL Technologies) according to the manufacture's protocol. Colony types were evaluated after 14 days of incubation at 37°C, 5% CO_2 by inverted light microscopy (Olympus).

Chemotaxis Assay

Chemotaxis was assessed using a microfluidic device as described previously (Yamahashi et al., 2015). In brief, polydimethylsiloxane devices were plasma treated and adhered to glass coverslips. Devices were coated with 10 μg/mL fibrinogen (Sigma) in PBS for 30 min at 37°C, 5% CO_2. The devices were blocked with 2% BSA-PBS for 30 min at 37°C, 5% CO_2, to block non-specific binding, and then washed twice with mHBSS. Cells were stained with calcein AM (Molecular Probes) in PBS for 10 min at room temperature followed by resuspension in modified Hank's balanced salt solution (mHBSS). Cells were seeded at 5×10^6/mL to allow adherence for 30 min before addition of chemoattractant. Either 1 μM fMLP (Sigma) or 11.25 μM IL-8 (R&D Systems) was loaded onto the devices. Cells were imaged for 45–90 min every 30 s on a Nikon Eclipse TE300 inverted fluorescent microscope with a 10× objective and an automated stage using MetaMorph software (Molecular Devices). Automated cell tracking analysis was done using JEX software (Warrick et al., 2016) to calculate chemotactic index and velocity.

Phagocytosis

Phagocytosis was assessed using pHrodo Green *E. coli* BioParticles Conjugate (Invitrogen) according to a modified manufacturer's protocol. pHrodo Green *E. coli* beads were resuspended in 2 mL of PBS and sonicated with an ultrasonicator 3 times (20% amplitude, 20 s on/10 s off). Beads per assay (100 μL) were opsonized by mixing with opsonizing reagent at a 1:1 ratio and incubated at 37°C for 1 h. Beads were washed 3 times mHBSS buffer by centrifugation at 4°C, 1,500 RCF for 15 min then final resuspension in mHBSS buffer. Beads were used immediately or stored at 4°C for several days. Cells (5×10^5) were resuspended in 100 μL of opsonized bead solution and incubated at 37°C or on ice for 1 h. Phagocytosis was stopped by placing all samples on ice. Analysis was carried out with Thermo Fisher Scientific Attune cytometer for fluorescent particles (509/533). Cells were gated based on granulocyte population, single cells, and live cells using propidium iodine.

Phagocytosis Imaging

Overnight culture of *Pseudomonas aeruginosa* was diluted 1:4 and grown in LB at 37°C for about 2 h, or until a 1:100 dilution, OD_{600} of 0.3 was reached. Empirically determined, cells were diluted to 1,000 colony-forming units/nL, in our hands OD_{600} 2.5. hiPSC-derived neutrophils (2^5) were mixed with 2^6 bacteria in mHBSS and then plated on a glass coverslip coated with 10 μg/mL fibrinogen and imaged at 37°C on a Nikon Eclipse TE300 inverted fluorescent microscope with a 60× oil immersion objective and an automated stage using MetaMorph software.

Measurement of Reactive Oxygen Species Production in Neutrophils

Floating cells (10^5) were plated in each well of a black 96-well plate on 10 μg/mL fibrinogen with 100 μL of mHBSS buffer in the presence of 10 ng/mL dihydrorhodamine 123. Cells were incubated for 30 min at 37°C/5% CO_2. PMA was added to a final concentration of 50 ng/mL or vehicle control DMSO was added to samples. Optimal reactive oxygen species production was determined by time course. Fluorescent measurements were taken of samples in triplicate or replicates of four on the Victor^3 V plate reader (Ex/Em 500/536).

NET Formation

hiPSC or primary blood neutrophils (2^5) were plated on 12-mm glass coverslips coated with 10 μg/mL fibrinogen and blocked

with 2% BSA. Cells were allowed to adhere for 30 min then treated, or not, with 100 nM PMA for 4 h. Assay was stopped with fixation by gentle addition of paraformaldehyde to 4% final concentration for 15 min. Cells were stained with human neutrophil elastase for 1 h in 5% goat serum then stained with Hoechst 33342 for 10 min before mounting. Cells were imaged using an LSM 800 Zeiss Airyscan 60× oil emersion lens.

Statistical Analysis

All the data reported as mean + SE. All the graphs and statistics were performed using GraphPad Prism software (GraphPad, San Diego, CA).

SUPPLEMENTAL INFORMATION

Supplemental Information can be found online at https://doi.org/10.1016/j.stemcr.2019.10.007.

AUTHOR CONTRIBUTIONS

VSB contributed to the concept, designed, conducted, analysed experiments, interpreted experimental data, made figures and contributed to paper writing. DAB performed neutrophil differentiation, chemotaxis assays, functional analysis, and made figures. KS made mmRNAs for the experiments, performed phagocytosis assay and hematoendothelial induction with mmRNA using transfection. LCK performed experimentation, and contributed to paper writing. IS and AH developed concept, supervised and lead the studies, analysed and interpreted data, and wrote the paper.

ACKNOWLEDGMENTS

We thank Matt Raymond for editorial assistance. This work was supported by funds from UW2020 Wisconsin Alumni Research Foundation (WARF; United States) program and National Institute of Health, United States (R01AI134749-01, R01HL142665, and P51 OD011106).

Received: May 1, 2019
Revised: October 10, 2019
Accepted: October 11, 2019
Published: November 7, 2019

REFERENCES

Badieyan, Z.S., and Evans, T. (2019). Concise review: application of chemically modified mRNA in cell fate conversion and tissue engineering. Stem Cells Transl. Med. *8*, 833–843.

Castagnola, E., Fontana, V., Caviglia, I., Caruso, S., Faraci, M., Fioredda, F., Garre, M.L., Moroni, C., Conte, M., Losurdo, G., et al. (2007). A prospective study on the epidemiology of febrile episodes during chemotherapy-induced neutropenia in children with cancer or after hemopoietic stem cell transplantation. Clin. Infect. Dis. *45*, 1296–1304.

Cavnar, P.J., Mogen, K., Berthier, E., Beebe, D.J., and Huttenlocher, A. (2012). The actin regulatory protein HS1 interacts with Arp2/3 and mediates efficient neutrophil chemotaxis. J. Biol. Chem. *287*, 25466–25477.

Chen, G., Gulbranson, D.R., Hou, Z., Bolin, J.M., Ruotti, V., Probasco, M.D., Smuga-Otto, K., Howden, S.E., Diol, N.R., Propson, N.E., et al. (2011). Chemically defined conditions for human iPSC derivation and culture. Nat. Methods *8*, 424–429.

Choi, K.D., Vodyanik, M.A., and Slukvin, I.I. (2009a). Generation of mature human myelomonocytic cells through expansion and differentiation of pluripotent stem cell-derived lin-CD34+CD43+CD45+ progenitors. J. Clin. Invest. *119*, 2818–2829.

Choi, K.D., Yu, J., Smuga-Otto, K., Salvagiotto, G., Rehrauer, W., Vodyanik, M., Thomson, J., and Slukvin, I. (2009b). Hematopoietic and endothelial differentiation of human induced pluripotent stem cells. Stem Cells *27*, 559–567.

Elcheva, I., Brok-Volchanskaya, V., Kumar, A., Liu, P., Lee, J.H., Tong, L., Vodyanik, M., Swanson, S., Stewart, R., Kyba, M., et al. (2014). Direct induction of haematoendothelial programs in human pluripotent stem cells by transcriptional regulators. Nat. Commun. *5*, 4372.

Fares, I., Chagraoui, J., Gareau, Y., Gingras, S., Ruel, R., Mayotte, N., Csaszar, E., Knapp, D.J., Miller, P., Ngom, M., et al. (2014). Cord blood expansion. Pyrimidoindole derivatives are agonists of human hematopoietic stem cell self-renewal. Science *345*, 1509–1512.

Garry, D.J. (2016). Etv2 is a master regulator of hematoendothelial lineages. Trans. Am. Clin. Climatol. Assoc. *127*, 212–223.

Gea-Banacloche, J. (2017). Granulocyte transfusions: a concise review for practitioners. Cytotherapy *19*, 1256–1269.

Graw, R.G., Jr., Herzig, G., Perry, S., and Henderson, E.S. (1972). Normal granulocyte transfusion therapy: treatment of septicemia due to gram-negative bacteria. N. Engl. J. Med. *287*, 367–371.

Gurlek Gokcebay, D., and Akpinar Tekgunduz, S. (2018). Granulocyte transfusions in the management of neutropenic fever: a pediatric perspective. Transfus. Apher. Sci. *57*, 16–19.

Hayashi, M., Pluchinotta, M., Momiyama, A., Tanaka, Y., Nishikawa, S., and Kataoka, H. (2012). Endothelialization and altered hematopoiesis by persistent Etv2 expression in mice. Exp. Hematol. *40*, 738–750.e11.

Hu, K., Yu, J., Suknuntha, K., Tian, S., Montgomery, K., Choi, K.D., Stewart, R., Thomson, J.A., and Slukvin, I.I. (2011). Efficient generation of transgene-free induced pluripotent stem cells from normal and neoplastic bone marrow and cord blood mononuclear cells. Blood *117*, e109–119.

Kang, H., Mesquitta, W.T., Jung, H.S., Moskvin, O.V., Thomson, J.A., and Slukvin, I.I. (2018). GATA2 is dispensable for specification of hemogenic endothelium but promotes endothelial-to-hematopoietic transition. Stem Cell Reports *11*, 197–211.

Kataoka, H., Hayashi, M., Nakagawa, R., Tanaka, Y., Izumi, N., Nishikawa, S., Jakt, M.L., Tarui, H., and Nishikawa, S. (2011). Etv2/ER71 induces vascular mesoderm from Flk1+PDGFRalpha+ primitive mesoderm. Blood *118*, 6975–6986.

Kuijpers, T.W., Tool, A.T., van der Schoot, C.E., Ginsel, L.A., Onderwater, J.J., Roos, D., and Verhoeven, A.J. (1991). Membrane surface antigen expression on neutrophils: a reappraisal of the use of surface markers for neutrophil activation. Blood *78*, 1105–1111.

Lachmann, N., Ackermann, M., Frenzel, E., Liebhaber, S., Brennig, S., Happle, C., Hoffmann, D., Klimenkova, O., Luttge, D.,

Buchegger, T., et al. (2015). Large-scale hematopoietic differentiation of human induced pluripotent stem cells provides granulocytes or macrophages for cell replacement therapies. Stem Cell Reports *4*, 282–296.

Li, L., Qi, X., Sun, W., Abdel-Azim, H., Lou, S., Zhu, H., Prasadarao, N.V., Zhou, A., Shimada, H., Shudo, K., et al. (2016). Am80-GCSF synergizes myeloid expansion and differentiation to generate functional neutrophils that reduce neutropenia-associated infection and mortality. EMBO Mol. Med. *8*, 1340–1359.

Lyman, G.H., and Poniewierski, M.S. (2017). A patient risk model of chemotherapy-induced febrile neutropenia: lessons learned from the ANC study group. J. Natl. Compr. Canc. Netw. *15*, 1543–1550.

Mesquitta, W.T., Wandsnider, M., Kang, H., Thomson, J., Moskvin, O., Suknuntha, K., and Slukvin, I.I. (2019). UM171 expands distinct types of myeloid and NK progenitors from human pluripotent stem cells. Sci. Rep. *9*, 6622.

Penchansky, L., Pirrotta, V., and Kaplan, S.S. (1993). Flow cytometric study of the expression of neutral endopeptidase (CD10/CALLA) on the surface of newborn granulocytes. Mod. Pathol. *6*, 414–418.

Powell, D., Tauzin, S., Hind, L.E., Deng, Q., Beebe, D.J., and Huttenlocher, A. (2017). Chemokine signaling and the regulation of bidirectional leukocyte migration in interstitial tissues. Cell Rep. *19*, 1572–1585.

Saeki, K., Saeki, K., Nakahara, M., Matsuyama, S., Nakamura, N., Yogiashi, Y., Yoneda, A., Koyanagi, M., Kondo, Y., and Yuo, A. (2009). A feeder-free and efficient production of functional neutrophils from human embryonic stem cells. Stem Cells *27*, 59–67.

Scapini, P., Marini, O., Tecchio, C., and Cassatella, M.A. (2016). Human neutrophils in the saga of cellular heterogeneity: insights and open questions. Immunol. Rev. *273*, 48–60.

Slukvin, I.I., Vodyanik, M.A., Thomson, J.A., Gumenyuk, M.E., and Choi, K.D. (2006). Directed differentiation of human embryonic stem cells into functional dendritic cells through the myeloid pathway. J. Immunol. *176*, 2924–2932.

Suknuntha, K., Tao, L., Brok-Volchanskaya, V., D'Souza, S.S., Kumar, A., and Slukvin, I. (2018). Optimization of synthetic mRNA for highly efficient translation and its application in the generation of endothelial and hematopoietic cells from human and primate pluripotent stem cells. Stem Cell Rev. *14*, 525–534.

Sumanas, S., and Choi, K. (2016). ETS transcription factor ETV2/ER71/Etsrp in hematopoietic and vascular development. Curr. Top. Dev. Biol. *118*, 77–111.

Sweeney, C.L., Teng, R., Wang, H., Merling, R.K., Lee, J., Choi, U., Koontz, S., Wright, D.G., and Malech, H.L. (2016). Molecular analysis of neutrophil differentiation from human induced pluripotent stem cells delineates the kinetics of key regulators of hematopoiesis. Stem Cells *34*, 1513–1526.

Trump, L.R., Nayak, R.C., Singh, A.K., Emberesh, S., Wellendorf, A.M., Lutzko, C.M., and Cancelas, J.A. (2019). Neutrophils derived from genetically modified human induced pluripotent stem cells circulate and phagocytose bacteria in vivo. Stem Cells Transl. Med. *8*, 557–567.

Valentini, C.G., Farina, F., Pagano, L., and Teofili, L. (2017). Granulocyte transfusions: a critical reappraisal. Biol. Blood Marrow Transpl. *23*, 2034–2041.

Warrick, J.W., Timm, A., Swick, A., and Yin, J. (2016). Tools for single-cell kinetic analysis of virus-host interactions. PLoS One *11*, e0145081.

Werfel, T., Sonntag, G., Weber, M.H., and Gotze, O. (1991). Rapid increases in the membrane expression of neutral endopeptidase (CD10), aminopeptidase N (CD13), tyrosine phosphatase (CD45), and Fc gamma-RIII (CD16) upon stimulation of human peripheral leukocytes with human C5a. J. Immunol. *147*, 3909–3914.

West, K.A., Gea-Banacloche, J., Stroncek, D., and Kadri, S.S. (2017). Granulocyte transfusions in the management of invasive fungal infections. Br. J. Haematol. *177*, 357–374.

Yamahashi, Y., Cavnar, P.J., Hind, L.E., Berthier, E., Bennin, D.A., Beebe, D., and Huttenlocher, A. (2015). Integrin associated proteins differentially regulate neutrophil polarity and directed migration in 2D and 3D. Biomed. Microdevices *17*, 100.

Yu, J., Hu, K., Smuga-Otto, K., Tian, S., Stewart, R., Slukvin, I.I., and Thomson, J.A. (2009). Human induced pluripotent stem cells free of vector and transgene sequences. Science *324*, 797–801.

Zarco, M.A., Ribera, J.M., Urbano-Ispizua, A., Filella, X., Arriols, R., Martinez, C., Feliu, E., and Montserrat, E. (1999). Phenotypic changes in neutrophil granulocytes of healthy donors after G-CSF administration. Haematologica *84*, 874–878.

Zhao, H., and Choi, K. (2017). A CRISPR screen identifies genes controlling Etv2 threshold expression in murine hemangiogenic fate commitment. Nat. Commun. *8*, 541.

Stem Cell Reports, Volume *13*

Supplemental Information

Effective and Rapid Generation of Functional Neutrophils from Induced Pluripotent Stem Cells Using ETV2-Modified mRNA

Vera S. Brok-Volchanskaya, David A. Bennin, Kran Suknuntha, Lucas C. Klemm, Anna Huttenlocher, and Igor Slukvin

Supplementary Information

V.S. Brok-Volchanskaya, D. A. Bennin, K. Suknuntha, L. Klemm, A. Huttenlocher, and I. Slukvin

Effective and rapid generation of functional neutrophils from induced pluripotent stem cells using ETV2 modified mRNA

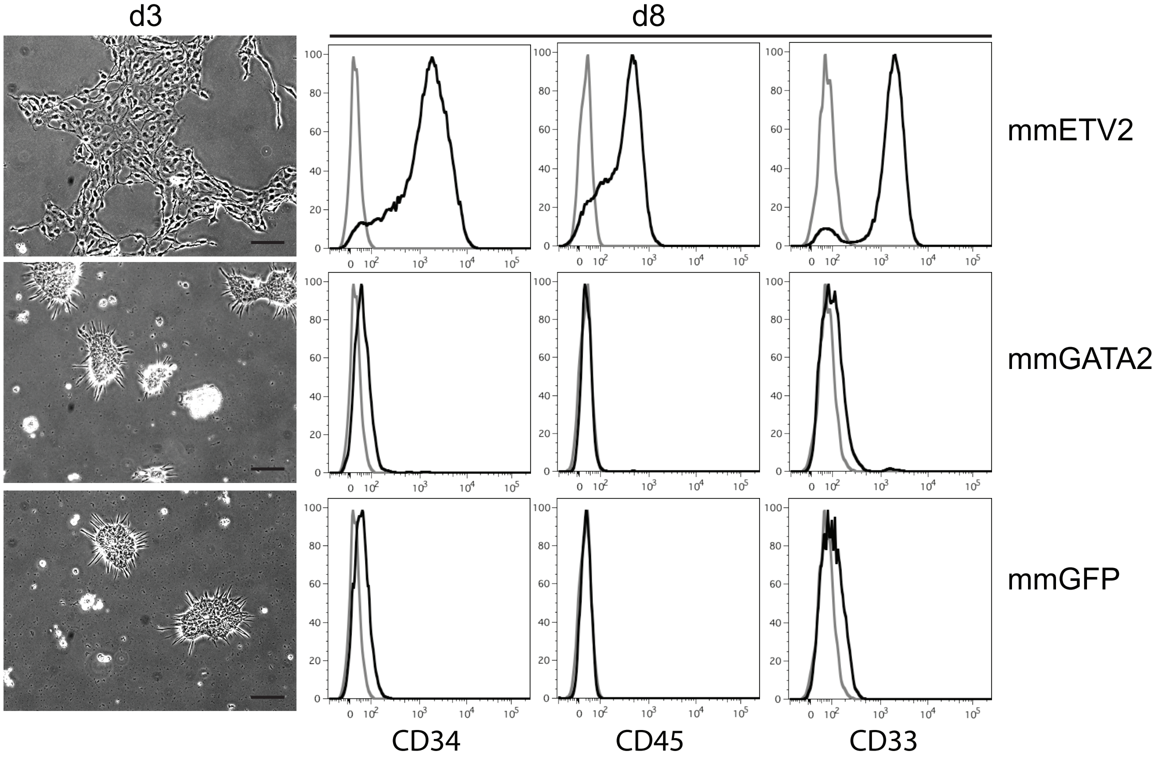

Supplementary Figure 1. Induction of myeloid progenitors is specific for ETV2 modified mRNA. iPSCs were transfected with ETV2, GATA2 or GFP modified RNA and cultured as outlined in Fig. 1A. Formation of $CD34^{+}CD33^{+}CD45^{+}$ myeloid progenitors was observed only in ETV2 mmRNA transfected cultures.

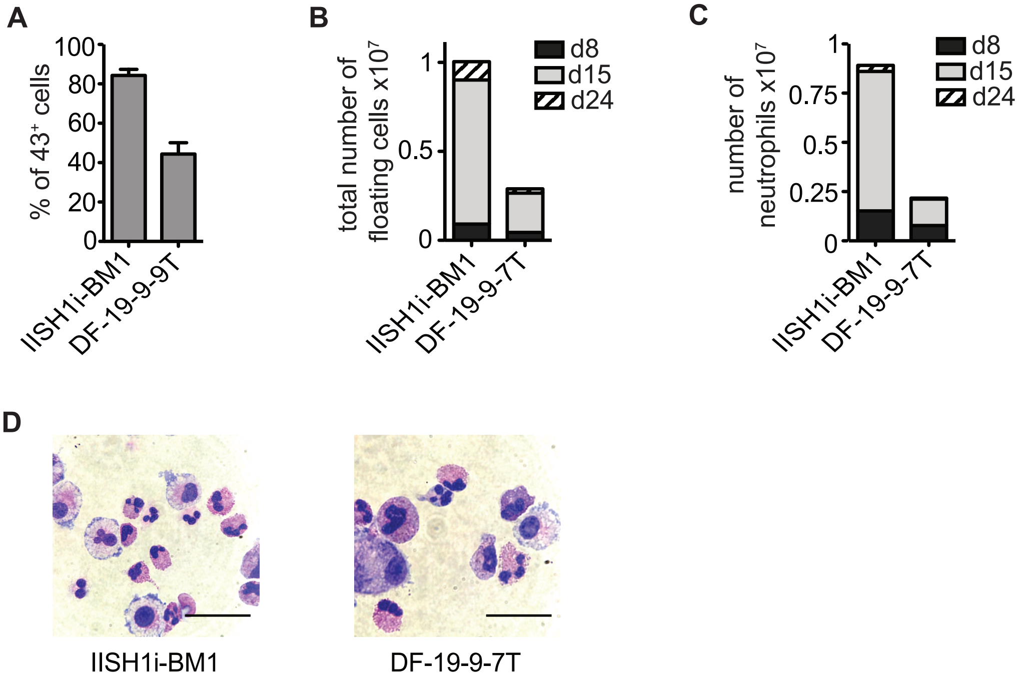

Supplementary Figure 2. Neutrophil production from IISH1i-BM9 and DF-19-7T iPSC lines using ETV2 modified mRNA. (A) Proportion of $CD43^+$ cells formed on day 8 of differentiation. (B) Total number of floating cells collected from differentiation cultures. (C) Total number of neutrophils produced in cultures. (D) Cytospins showing the morphology of the generated neutrophils.

Supplementary Video 1-4

Video 1-Fig 4a hiPSC derived neutrophils random migration.

Video 2-Fig 4a hiPSC derived neutrophils chemotaxing to fMLP.

Video 3-Fig 4a hiPSC derived neutrophils chemotaxing to IL8.

Video 4-Fig 4c hiPSC derived neutrophils migrating and phagocytosing *Pseudomonas aeruginosa.*

Supplementary Table 1. Antibodies used in this study.

Antigen	Conjugate	Source	Cat No.
CD10	BV510	BD Biosciences	563032
CD11b	APC	BD Biosciences	550019
CD11b	PE	Life Technologies	12-0118-42
CD15	BV711	BD Biosciences	563142
CD16	FITC	Life Technologies	11-0168-42
CD16	APC	Life technologies	17-0168-42
CD16	PE-Cy7	BD Biosciences	557744
CD33	PE-Cy5	Biolegend	303406
CD34	PE-Vio770	Miltenyi Biotec	130-100-844
CD34	FITC	BD Bioseceinces	555821
CD41a	APC	BD Bioseceinces	559777
CD43	VioBlue	Miltenyi Biotec	130-097-373
CD45	PE	Miltenyi Biotec	130-113-118
CD45	PE-Vio770	Miltenyi Biotec	130-110-634
CD45	APC-Vio770	Miltenyi Biotec	130-110-773
CD66b	PE	BD Biosciences	561650
CD144	PE-Vio770	Miltenyi Biotec	130-100-720
CD182	PE	Invitrogen	12-1829-42
CD235a	APC	BD Bioseceinces	551336
Lactoferrin	PE	Life Technologies	GIC206
MPO	FITC	Invitrogen	11-1299-42
Ghost red	780	Tonbo Biosciences	13-0865-T100
